# Safety Evaluation of Reinforced Concrete Structures Using Multi-Source Fusion Uncertainty Cloud Inference and Experimental Study

**DOI:** 10.3390/s23208638

**Published:** 2023-10-22

**Authors:** Zhao Liu, Huiyong Guo, Bo Zhang

**Affiliations:** School of Civil Engineering, Chongqing University, Chongqing 400045, China; 202116021227t@cqu.edu.cn (Z.L.); 202116021116t@cqu.edu.cn (B.Z.)

**Keywords:** structural health monitoring, safety evaluation, damage detection, uncertainty inference, reinforced concrete structure

## Abstract

Structural damage detection and safety evaluations have emerged as a core driving force in structural health monitoring (SHM). Focusing on the multi-source monitoring data in sensing systems and the uncertainty caused by initial defects and monitoring errors, in this study, we develop a comprehensive method for evaluating structural safety, named multi-source fusion uncertainty cloud inference (MFUCI), that focuses on characterizing the relationship between condition indexes and structural performance in order to quantify the structural health status. Firstly, based on cloud theory, the cloud numerical characteristics of the condition index cloud drops are used to establish the qualitative rule base. Next, the proposed multi-source fusion generator yields a multi-source joint certainty degree, which is then transformed into cloud drops with certainty degree information. Lastly, a quantitative structural health evaluation is performed through precision processing. This study focuses on the numerical simulation of an RC frame at the structural level and an RC T-beam damage test at the component level, based on the stiffness degradation process. The results show that the proposed method is effective at evaluating the health of components and structures in a quantitative manner. It demonstrates reliability and robustness by incorporating uncertainty information through noise immunity and cross-domain inference, outperforming baseline models such as Bayesian neural network (BNN) in uncertainty estimations and LSTM in point estimations.

## 1. Introduction

With the continuous improvement in social infrastructure construction techniques, structural safety is being confronted with the challenges of large volumes, complex structures, and harsh environments [1,2,3]. This can result in complications, such as delayed structural damage diagnoses and inaccurate safety evaluations. A current focus in structural health monitoring research is exploring the correlation between monitoring data and structure service performance for quantitative evaluations based on existing safety evaluation methods.

Reinforced concrete (RC) has a wide range of applications in infrastructure, such as bridges and buildings. Over time, concrete structures may exhibit degraded stiffness and reduced bearing capacity due to cracking, corrosion of reinforcements, and other types of damage [4]. Moradi et al. [5] carried out four-point bending tests on RC slabs to extract the energy of the received signals to assess the loss of signal energy due to structural damage, verifying the applicability of ultrasound monitoring in assessments of damage in RC structures. Farhidzadeh et al. [6] proposed a quantitative grading method for RC components based on the damage index of the residual cracking state. It was shown that the proposed method could more accurately assess the damage level of components and determine their relative stiffness loss through low-cycle reciprocating loading of two large RC shear walls. Xiao et al. [7] introduced a damage detection method that considers the shear deformation of slender beam frame structures and precisely identifies damage by adjusting the structure parameters to match the measured displacements. Asjodi et al. [8] developed a probabilistic framework to analyze the spatial distribution of cracking and crushing in RC shear walls. To demonstrate its validity, they carried out a comprehensive probabilistic spatial analysis of RC shear walls subjected to cyclic loading. In summary, the intrinsic relationship between damage states and the structural performance of RC structures is significant, reflecting changes in properties such as structural stiffness and load-carrying capacity.

The analytical hierarchy process (AHP) is essential for establishing an evaluation system within a comprehensive evaluation method by decomposing the evaluation object into multiple levels, such as the objective, guideline, and program levels [9]. The key is to solve the weight coefficients of each level. However, this process depends entirely on the experience of experts to construct the judgment matrix; therefore, subjective problems remain that must be addressed by studying them in depth.

The fuzzy synthesis method is based on the theory of fuzzy mathematics, in which uncertain information is quantitatively represented and the evaluation results obtained with the help of a generalized fuzzy synthesis operation [10]. Tesfamariam et al. [11] developed a modeling approach based on a fuzzy rule knowledge base to assess the seismic vulnerability of buildings through fuzzy set theory. Sun et al. [12] proposed a fuzzy theory-based model to assess the impact of explosion accidents (due to the use of hazardous materials) on bridge safety, established a hierarchical structure for bridge explosion disaster risk assessment, and validated the reasonableness of the proposed method by using relevant cases. Through the use of the fuzzy synthesis method, the fuzzy characteristics of things in the safety evaluation process can be better identified. However, there needs to be more objectivity in the determination criteria for the elements of the evaluation model, whose output is in the stage of qualitative determination [13]. Contrastingly, the gray theoretical models make it possible to perform quantitative analyses of the dynamic development process of a system to identify the primary and secondary factors that influence the state of development of the system [14]. This allows such models to effectively undertake evaluations using few data samples and to better reflect the uncertainty in knowledge, which is random [15]. Zhao et al. [16] considered the complex factors affecting the fatigue life of steel wire ropes by applying the gray theory to small-sample fatigue life data to effectively improve life prediction accuracy.

The emphasis on the static reaction of a structure is evident in the techniques employed for damage detection [17] and sensor placement [18] for SHM. Xiao et al. [19] developed the stiffness separation method to divide the global stiffness into sub-stiffness matrices, making it possible to undertake precise evaluations of truss damage in space using static responses. On the other hand, the reliability theory is an evaluation method based on probabilistic statistics that establishes the uncertainty relationship between the loads and resistances in a structure and uses the reliability index or probability of failure to assess the safety status of that structure [20]. Guo et al. [21] developed a traffic load model based on monitoring data obtained from a vehicle dynamic weighing system, which was combined with probabilistic finite element (FE) analysis and applied to a fatigue reliability evaluation of an in-service bridge. To summarize, at the core of evaluation methods based on reliability theory is the need to determine the analytical formulas for loads and resistances, with particular focus on solving the systematic failure modes of complex structures.

With the rapid development of artificial intelligence technology, structural safety evaluations are shifting from traditional, model-driven modes to data-driven modes [22]. The artificial neural network (ANN) is gradually being adopted by the civil engineering community due to its advantages of not requiring manual extraction of features and its superior ability to establish the mapping between inputs and outputs to enable end-to-end evaluations [23,24]. Shen et al. [25] proposed a deep neural network-based structural safety state evaluation method that uses acceleration spectra and structural safety state as the model inputs and outputs, respectively. They verified the validity and accuracy of this approach through a five-layer RC framework structure. Liu et al. [26] introduced a risk warning model based on a convolutional neural network according to the uncertainty characteristics of risk factors in engineering. Indeed, neural network models tend to require many samples for training, and the scarcity of such data is an ongoing issue [27].

Comparatively, via cloud theory, which is based on probability theory and fuzzy theory, the concept of an affiliation cloud was proposed; this is a novel mathematical tool and decision-making instrument for solving the quantification of uncertainty knowledge [28,29,30]. Cloud theory, as an artificial intelligence algorithm, is currently being widely used in several fields, including data mining and knowledge discovery [31], decision analysis [32], mechanical diagnosis [33], and safety evaluation [34]. Zhou et al. [35] proposed a cloud model method with entropy-containing weights for the classification prediction of rock bursts, verifying the validity by zoning 209 sets of rock burst samples from underground rock engineering. Wang et al. [36] suggested a new connected cloud model for the multiple uncertainties and distribution characteristics of slope stability evaluation indexes. Lin et al. [37] combined variable-weight theory with cloud theory to construct a new computational model for assessing the construction risk of karst tunnels with respect to the risk evaluation of tunnel construction safety regarding sudden water hazards.

Based on the above, a new structural safety evaluation method, named multi-source fusion uncertainty cloud inference (MFUCI), is proposed in this paper. It may be used to assess the uncertainty caused by initial defects and monitoring errors in practical engineering structures. The method focuses on studying the relationship between characterization condition indexes and structural performance in order to quantitatively evaluate the structural health status of RC structures, from the component level to the whole structural level. An evaluation was performed through damage experiments on RC components and FE simulations of single-story RC frame structures. The proposed method facilitates comprehensive safety evaluations by considering the individual components and the whole structure separately.

Regarding the rest of this paper, Section 2 describes cloud theory. Section 3 describes the mathematical theory, architectural design, and evaluation index system of the proposed method. Section 4 and Section 5 present the numerical simulations and experimental studies used to evaluate and validate the performance of all aspects of the proposed method at the component and structural levels, respectively. The conclusions are reported in Section 6.

## 2. Cloud Theory Basics

### 2.1. Cloud Model

Assume that *U* (Universe) is a quantitative domain described by exact values and *C* (Concept) is a qualitative concept on a quantitative domain. There is a quantitative value x∈U, which is a random realization on qualitative concept *C*, and the certainty degree u(x)∈[0, 1] of *x* on *C* is a random number with a stable tendency. Synthetically, the distribution of *x* over theoretical domain *U* is a cloud model, as shown below:

(1) u:U→[0, 1]

(2) ∀x∈U,x→u(x)

where each *x* is a cloud drop, denoted as *drop*(*x*, *u*(*x*)). The numerical characteristics of clouds include expectation, entropy, and super-entropy. These characteristics can describe the overall characteristics of cloud models so that the interconversion between qualitative and quantitative parameters can be realized [38]. The three numerical characteristics are defined as follows:

1. Expectation (*Ex*): The mathematical expectation of the distribution of cloud drops on domain space *U*, which can reflect the information center value of the corresponding fuzzy concept. It is also the central location of the range covered by the affiliation cloud map, i.e., theoretical domain value *x* when certainty degree *u*(*x*) = 1;
2. Entropy (*En*): Reflects the degree of dispersion of cloud drops and the randomness of the qualitative concept; the higher the entropy value, the more extensive the range of values of the qualitative concept in the quantitative domain, which is reflected in the cloud diagram as the “span” of the cloud;
3. Hyper entropy (*He*): Hyper entropy is a measure of uncertainty about entropy, i.e., the entropy of entropy, which captures the degree of coalescence of cloud drops. It reflects the degree of randomness of quantitative value *x* on qualitative concept *C*.

The certainty degree *u*(*x*) for qualitative concept *C* is a one-to-many mapping relationship when given a quantitative *value, x, which applies over theoretical* domain *U*. The generation of cloud drops is random, which is an uncertain representation of qualitative concept *C*. For the normal cloud model, the cloud drop groups located in different zones contribute differently to the qualitative concept. The contribution (∆*C*) of cloud drop group ∆*x* to the qualitative concept on any small interval in a given theoretical domain is:

(3) $$\Delta C\approx \frac{u(\Delta x)\Delta x}{\sqrt{2\pi }En}$$

The total contribution of all elements to the qualitative concept is:

(4) $$C=\frac{\int_{-\infty }^{+\infty }u_{r}(x)dx}{\sqrt{2\pi }En}=\frac{\int_{-\infty }^{+\infty }\exp\left(-(x-Ex)/2En^{2}\right)dx}{\sqrt{2\pi }En}=1$$

Meanwhile:

(5) $$\frac{1}{\sqrt{2\pi }En}\int_{Ex-3En}^{Ex+3En}u_{T}(x)dx=99.74\%$$

Synthetically, Equation (5) indicates that 99.74% of the cloud drops contributing to qualitative concept C fall predominantly within interval [*Ex*−3*En*, *Ex*−3*En*], referred to as the 3*En* principle [39]. Within this interval, the model regards cloud drops to be valid, while a few anomalies and outlier point drops outside this interval are negligible [40]. Based on this property, there is computational derivation of cloud model characteristic parameters within the threshold interval, which will be reported in Section 3.2. The cloud model concept is usually represented as (*Ex*, *En*, *He*). The different contributions of cloud drops are classified as shown in Table 1.

### 2.2. Normal Cloud

The cloud generator is an essential foundation for generating, transforming, and mapping cloud models. The cloud generators can be divided into forward and backward cloud generators according to the functions to be achieved. Both can realize the interconversion between qualitative concepts and quantitative data, which is a mutual inverse process.

The forward cloud generator obtains quantitative value *x* of *N* cloud drops and certainty degree *u*(*x*) for that cloud drop concerning the qualitative concept by inputting the numerical characteristics of the cloud model (*Ex*, *En*, *He*) and the number of cloud drops *N*, which can be expressed as *drop*(*x*, *u*(*x*)). Synthetically, the forward cloud generator enables the range and distribution of quantitative data from the qualitative information of the concept expression. The specific steps of the forward cloud generator algorithm are as follows:

1. The inputs represent the numerical characteristics (*Ex*, *En*, *He*) of qualitative concept *C* and number of cloud drops *N*;
2. Generate a normal random number $\varphi =\mathrm{norm}(En,He^{2})$ with *En* as the expected value and *He*^2^ as the variance;
3. Generate a normal random number $x_{0}=\mathrm{norm}(En,\varphi^{2})$ with *Ex* as the expectation and $\varphi^{2}$ as the variance;
4. Calculate the certainty degree $u_{0}=\exp[-{\left(x_{0}-Ex\right)}^{2}/(2\varphi^{2})]$ and obtain a cloud drop $drop(x_{0},u_{0})$;
5. Repeat steps 2 to 4 until *N* cloud drops are generated.

The backward cloud generator algorithm is based on the statistical principle of converting a certain amount of known sample data into a qualitative concept expressed in terms of three major numerical characteristics: expectation, entropy, and hyper entropy. Therefore, it can be regarded as the inverse process of the forward cloud generator. For the input quantitative values, the numerical characteristics of cloud drops satisfying the normal distribution law are obtained by the backward cloud generator. In turn, the uncertainty conversion process from quantitative to qualitative concepts is realized. The input sample set is $X=\{x_{1},x_{2},\ldots ,x_{n}\}$ and the sample mean is calculated as follows:

(6) $$\bar{X}=\frac{1}{n}\sum_{i=1}^{n}x_{i}$$

First-order sample absolute central moment:

(7) $$M=\frac{1}{n}\sum_{i=1}^{n}\left|x_{i}-\bar{X}\right|$$

Sample variance:

(8) $$S^{2}=\frac{1}{n-1}\sum_{i=1}^{n}{\left(x_{i}-\bar{X}\right)}^{2}$$

Next, numerical characteristic values $\hat{E}x$, $\hat{E}n$, and $\hat{H}e$ are calculated separately:

(9) $$\hat{E}x=\bar{X}$$

(10) $$\hat{E}n=\sqrt{\frac{\pi }{2}}\times \frac{1}{n}\sum_{i=1}^{n}\left|x_{i}-Ex\right|=\sqrt{\frac{\pi }{2}}M$$

(11) $$\hat{H}e=\sqrt{S^{2}-En^{2}}$$

## 3. Multi-Source Fusion Uncertainty Cloud Inference

This section describes the proposed theoretical model for structural safety evaluation, named multi-source fusion uncertainty inference (MFUCI). It is dedicated to establishing the relationship between the multi-source condition index and structural performance level, making it possible to grade and quantify structural safety evaluations.

### 3.1. Condition Cloud Generator

The cloud model characteristic parameters are used as a basis to generate a cloud map which is capable of characterizing the certainty degree, where specific quantitative values (*x*) or given specific certainty degree *u* can be used as different input conditions. Further, the forward cloud generator is divided into antecedent and consequent cloud generators, with a combination of the two serving as an essential basis for cloud model uncertainty inference.

**Figure 1:**
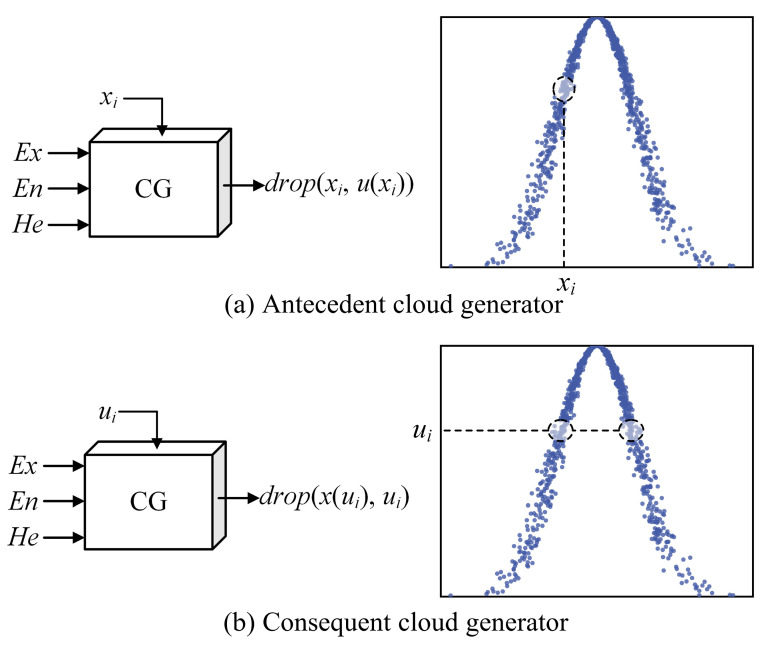
Principles of antecedent and consequent cloud generators.

A schematic diagram of the antecedent and consequent cloud generators is shown in Figure 1. When quantification *x_i_* in theoretical domain *U* is given, certainty degree *u*(*x_i_*) for quantification *x_i_* over qualitative concept *C* is generated by the forward cloud generator, which is called the antecedent cloud generator (X-condition generator). The generator takes values with a certain randomness, since *u*(*x_i_*) is generated randomly under the law of conforming to normal distribution. The consequent cloud generator (Y-conditional generator) refers to the process of calculating the quantitative value *x*(*u_i_*) that satisfies certainty degree *u_i_* on the qualitative concept by the forward cloud generator for certainty degree $u_{i}\in [0,\;1]$. Similarly, each realization of quantitative value *x*(*u_i_*) has uncertainty.

### 3.2. Multi-Source Fusion Generator

**Figure 2:**
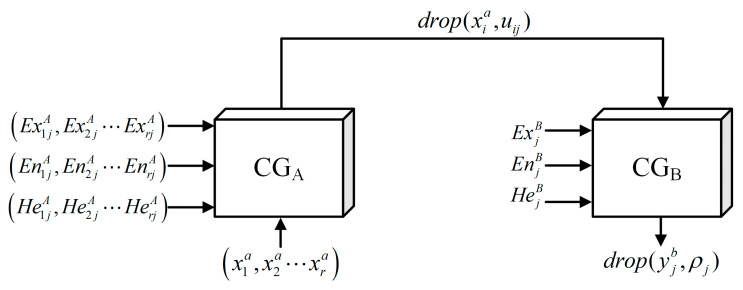
Multi-source fusion generator.

Establishing a qualitative rule base is particularly critical for cloud-theoretic uncertainty inference. The qualitative rule base usually comprises an antecedent and a consequent component, represented as the single conditional rule. By connecting the antecedent cloud generator and the consequent cloud generator, a qualitative rule generator based on the cloud model is formed, enabling the whole process of cloud inference. In evaluating structural safety states, it is necessary to frequently consider the influence of multiple index factors. Therefore, this paper proposes MFUCI, which takes the security evaluation condition index system as the input term of the antecedent and the performance level in different states of the structure as the output state of the consequent. In this way, the mapping relationship between the condition index and the performance level may be investigated. By applying this approach, safety evaluations of structures in an unknown damage state may be realized. Compared with the single-condition inference structure, the rule antecedent of MFUCI is constructed by the fusion of multi-source indexes and connected to a single consequent cloud generator, whose implementation principle is shown in Figure 2.

First, a sample representation corresponds to *r* condition indexes as $x_{i}^{a}$, which is input to the multi-source antecedent cloud generator for transformation into qualitatively conceptualized cloud numerical characteristics $\left(Ex_{ij}^{A},En_{ij}^{A},He_{ij}^{A}\right)$ under *s* grade intervals.

Next, the multi-source joint certainty degree is computed as:

(12) $$u_{ij}=\exp\left(-\frac{{\left(x_{i}^{a}-Ex_{ij}^{A}\right)}^{2}}{2{\left(\varphi_{ij}^{A}\right)}^{2}}\right)$$

(13) $$\rho_{j}=\prod_{i=1}^{n}u_{ij}$$

where $u_{ij}$ is the sample certainty of $x_{i}^{a}$ under *j*-th grade interval, denoted by $drop(x_{i}^{a},u_{ij})$, $\varphi_{ij}^{A}$ denotes a normal random number with $En_{ij}^{A}$ as the expectation and $He_{ij}^{A}$ as the standard deviation, and $\rho_{j}$ is the multi-source joint certainty degree under the *j*-th grade interval.

Then, $drop(x_{i}^{a},u_{ij})$ corresponds to the consequent cloud numerical characterizations as $\left(Ex_{j}^{B},En_{j}^{B},He_{j}^{B}\right)$, which is transformed into a quantitative value of the quantitative concept, calculated as:

(14) $$x_{ij}^{b}=\left\{\begin{array}{c} Ex_{j}^{B}+\varphi_{ij}^{B}\sqrt{-2\ln(u_{ij})}x_{i}^{a}>Ex_{ij}^{A}\\ Ex_{j}^{B}-\varphi_{ij}^{B}\sqrt{-2\ln(u_{ij})}x_{i}^{a}\leq Ex_{ij}^{A} \end{array}\right.$$

where $\varphi_{ij}^{B}$ denotes a normal random number with $En_{j}^{B}$ as the expectation and $He_{j}^{B}$ as the standard deviation.

Further, the fusion of the quantitative values under each condition index results in a consequent output value for the *j*-th grade interval obtained. It is calculated as:

(15) $$y_{j}^{b}=\frac{\sum_{i=1}^{r}x_{ij}^{b}u_{ij}}{\sum_{i=1}^{r}u_{ij}}$$

Finally, the inference value of the multi-source fusion via the precision processing is output:

(16) $$f_{out}=\frac{\sum_{j=1}^{s}y_{j}^{b}\rho_{j}}{\sum_{j=1}^{s}\rho_{j}}$$

In general, the proposed MFUCI is applied to structural safety evaluations. It constitutes different qualitative rules for the mapping relationship between the response values of each condition index and the structural performance level under different damage states of the structure. For the input response value of the condition index, which can be considered to be cloud drops without deterministic information, the multi-source fusion generator can be used to obtain the consequent cloud drops with deterministic information. Finally, the specific output value is obtained via precision processing. The uncertainty reasoning of the cloud model reflects the existence of an “IF–THEN” mapping relationship, which means that the response value of the monitoring index of the structure in a particular safety state has a corresponding health value. Since there is uncertainty in determining the threshold values of each index interval, the cloud model is utilized to handle the uncertainty and ambiguity well. The damage condition indexes and health degree corresponding to each grade interval are represented by the three numerical features $\left(Ex_{ij}^{A},En_{ij}^{A},He_{ij}^{A}\right)$ of the cloud model. Taking interval $\left(\eta_{ij}^{L},\eta_{ij}^{R}\right)$ as an illustration, expectation $Ex_{ij}^{A}$ is determined by:

(17) $$Ex_{ij}^{A}=\frac{\left(\eta_{ij}^{L}+\eta_{ij}^{R}\right)}{2}$$

According to the “3*En*” principle for cloud models, as described in Section 2.1, the interval threshold $\left(\eta_{ij}^{L},\eta_{ij}^{R}\right)$ satisfies the following:

(18) $$\left\{\begin{array}{c} \eta_{ij}^{L}=Ex_{ij}^{A}-3En_{ij}^{A}\\ \eta_{ij}^{R}=Ex_{ij}^{A}+3En_{ij}^{A} \end{array}\right.$$

From Equation (18), the entropy $En_{ij}^{A}$ is determined by:

(19) $$En_{ij}^{A}=\frac{\eta_{ij}^{R}-\eta_{ij}^{L}}{6}$$

The hyper entropy $He_{ij}^{A}$ is determined by:

(20) $$He_{ij}^{A}=\frac{En_{ij}^{A}}{\alpha }$$

where α is the amplitude modulation factor of the hyper entropy. In summary, the numerical characteristics of each evaluation index cloud model and the corresponding security level cloud model characteristic values constitute several rules. This results in a rule base consisting of several “IF–THEN” qualitative rules with qualitative concepts. Specifically, the “IF–THEN” rule is a typical rule used in fuzzy systems. The IF part is the antecedent, also known as the premise, of the inference system, while the THEN part is the consequent [41]. In this case, the structural response of the monitoring index serves as the antecedent of the inference system, while the corresponding health values serve as the consequent of this system in constructing the comprehensive inference system.

### 3.3. Evaluation Index System

To achieve efficient, fast, and intelligent safety evaluations of structural service performance, this paper focuses on the multi-source data that can be directly monitored by sensors to finalize the creation of the evaluation index system. Structural damage condition indexes are often established based on mechanical parameters, which are the response characteristics of a structure after being subjected to various influencing factors, such as stress, strain, displacement, inclination, and others. Accordingly, the proposed study considers the easily and directly monitored condition index as an essential structural safety performance evaluation basis. It expresses the monitoring-based multi-source condition index as follows:

(21) $$\Lambda ={\left[\Lambda_{1},\Lambda_{2},\ldots ,\Lambda_{r}\right]}^{T}$$

where Λ denotes the damage parameters for structural safety evaluations and $\Lambda_{i}$ characterizes the specific evaluation indexes in safety evaluations, such as strain, deflection, stress, etc.

The current health degree of a structure relative to its intact state is described in reference to a Chinese design code (JT/T 1037-2022) [42]. On this basis, this paper further makes the quantification of the health degree index for the practical situation of the research object:

(22) $$H_{i}=\frac{h_{i}}{h_{0}}$$

where *h_i_* denotes the actual performance level of the structure corresponding to a particular state, *h*_0_ denotes the established performance level of the structure in its initial state, and *H_i_* denotes the health degree of the structure in a particular state. According to the definition, *H_i_* can range from 0 to 1. When *H_i_* = 1, the structure is in an intact state.

Further, health index *H_i_* is used to reasonably divide the corresponding safety level, determining the interval thresholds of each damage condition index and health index. Assuming that evaluation model *M* based on MFUCI is divided into a total of *s* safety state levels and *r* damage condition indexes, the evaluation system model can be expressed as:

(23) $$M=\left[\left.\begin{array}{c} H\\ \Lambda_{1}\\ \Lambda_{2}\\ \vdots \\ \Lambda_{r} \end{array}\right|\;\begin{array}{cccc} \left(H_{1}^{L},H_{1}^{R}\right) & \left(H_{2}^{L},H_{2}^{R}\right) & \cdots & \left(H_{s}^{L},H_{s}^{R}\right)\\ \left(\eta_{11}^{L},\eta_{11}^{R}\right) & \left(\eta_{12}^{L},\eta_{12}^{R}\right) & \cdots & \left(\eta_{1s}^{L},\eta_{1s}^{R}\right)\\ \left(\eta_{21}^{L},\eta_{21}^{R}\right) & \left(\eta_{22}^{L},\eta_{22}^{R}\right) & \cdots & \left(\eta_{2s}^{L},\eta_{2s}^{R}\right)\\ \vdots & \vdots & \vdots & \vdots \\ \left(\eta_{r1}^{L},\eta_{r1}^{R}\right) & \left(\eta_{r2}^{L},\eta_{r2}^{R}\right) & \cdots & \left(\eta_{rs}^{L},\eta_{rs}^{R}\right) \end{array}\right]$$

where $\left(H_{j}^{L},H_{j}^{R}\right)$ denotes the health degree interval threshold corresponding to the *j*-th safety level and $\left(\eta_{ij}^{L},\eta_{ij}^{R}\right)$ denotes the interval threshold of evaluation index $\Lambda_{i}$ at the *j*-th safety level.

### 3.4. Architecture of MFUCI

**Figure 3:**
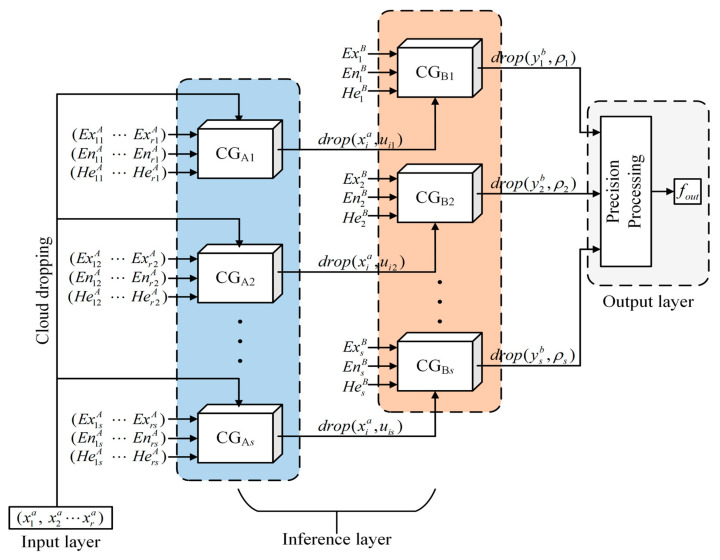
The basic architecture of the proposed MFUCI.

The MFUCI is constructed in the form shown in Figure 3. According to the implementation principle of the cloud model uncertainty inference method, the MFUCI can be divided into four parts: an input layer, cloud dropping, an inference layer, and an output layer. The meaning of each component is as follows:

1. Input layer: Monitoring-based damage condition index data for a given safety state of the structure;
2. Cloud dropping: The data vectors in the input layer are mapped to cloud titer values with no deterministic information and are then fed into the multi-source fusion generator for inference;
3. Inference layer: According to the principle of the multi-source fusion generator algorithm, each mapping rule between the existence of the damage condition index and the health degree in the qualitative rule base corresponds to the generator;
4. Output layer: The cloud drops that have deterministic information output from the antecedent cloud generator in the inference layer are refined to output the final structural health values to complete the inference process.

## 4. Numerical Simulation of RC Frame

This section focuses on a single-story RC structure in our investigation of damage progression under low-cycle cyclic loading. The focus is on quantifying the structural safety state and validating the effectiveness of the proposed research in conducting safety evaluations at the structural level.

### 4.1. Specimen and FE Parameters

**Figure 4:**
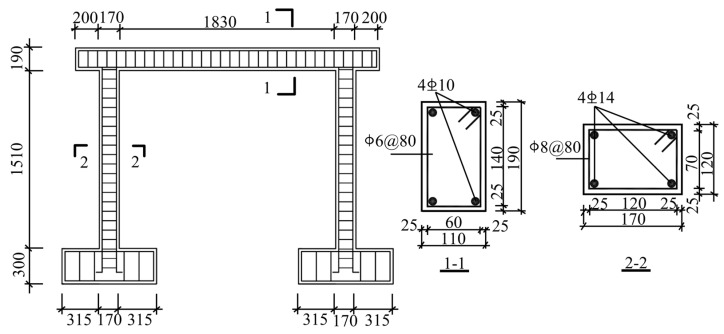
RC frame reinforcement diagram.

The structure is based on a one-bay frame in an industrial building with a column grid of 6 m × 6 m and a floor height of 5.1 m, designed and fabricated as a one-span, one-story RC frame specimen at a 1/3 scale for low-cycle reciprocating loading tests [43]. In this paper, the damage process of the specimen under horizontal reciprocating load is studied using a numerical simulation and the relevant specimen parameters. An RC frame reinforcement diagram is shown in Figure 4.

The compressive strength of concrete is C30, and the constitution adopts the concrete damaged plasticity (CDP) model, which can not only simulate the nonlinear behavior of concrete materials, such as stiffness degradation and other properties, but can also consider the distribution and development of damage at the macroscopic level [44]. The tensile and compressive damage factors of concrete are calculated according to the energy equivalent damage principle proposed by Sidiroff to simulate both the tensile and compressive damage behaviors of concrete materials in terms of plastic deformation [45]. The Clough correction model, which considers load-bearing capacity degradation [46], is adopted for the rebars to replace the degradation of strength and stiffness due to bond slip between concrete and rebar due to the degradation of rebar reloading stiffness. To realistically simulate the stresses of the RC structure and the convergence of FE, the rebar and concrete units are each separately modeled and meshed. Specifically, a positive hexahedral reduced integration solid element (C3D8R) is employed for concrete, while a truss element (T3D2) is utilized for rebar. The top surface of the column end is coupled to facilitate the application of vertical concentrated force through the coupling constraint. Similarly, the bottom of the structural base beam is fixed to apply a horizontal load on the left side of the horizontal beam with the displacement-controlled loading method.

### 4.2. Damage Quantification

**Figure 5:**
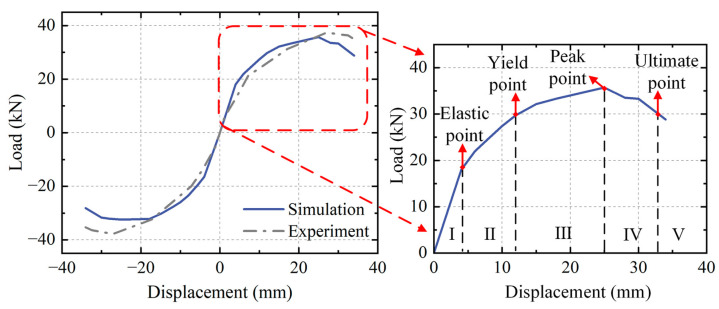
Comparison between simulation and experimental load–displacement skeleton curves and performance point segmentation.

The alignment between the extracted load–displacement skeleton curve and the experimental results is shown in Figure 5. A certain deviation is observed between the simulated results and the experimental skeleton curve due to factors such as the material properties and boundary conditions. The initial stiffness is relatively consistent, with the simulated peak load being slightly smaller than the experimental value. However, the FE model, overall, adequately captures the entire process of damage evolution in the model. The load–displacement skeleton curve represents the structural performance level at different stages. In this study, the different levels of structural damage are classified by extracting four characteristic points from the skeleton curve: the elastic point, yield point, peak point, and ultimate point. The health condition of the structure is categorized into five safety levels: intact, minor damage, moderate damage, severe damage, and failure.

**Figure 6:**
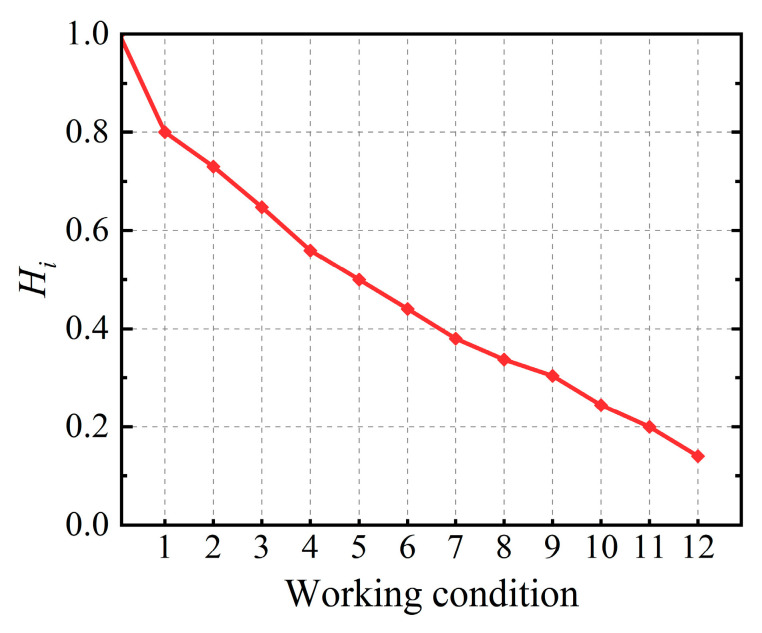
The health degree quantization values corresponding to each working condition of the RC frame.

The elastic point corresponds to a condition where there is only minor tensile damage on the surface of the component, with no significant compressive damage. The yield point is determined using the secant stiffness method and serves as the critical point between minor and moderate damage. The ultimate point is defined as the point at which the load capacity decreases to 85%; exceeding this point is an indication of structural failure [47]. The degradation of the secant stiffness of RC structures under low-cycle reciprocating loading characterizes a structural failure process and serves as a global damage index for the structure [48,49]. Therefore, in conjunction with Equation (22), the structural health degree of this case is specified as:

(24) $$K_{s,i}=\frac{F_{i}^{+}-F_{i}^{-}}{\Delta_{i}^{+}-\Delta_{i}^{-}}$$

(25) $$H_{i}=\frac{K_{s,i}}{K_{s,0}}$$

where *K*_s,*i*_ denotes the secant stiffness under the *i*-th cyclic loading, *K*_s,0_ denotes the linear stiffness in the intact condition of the structure, and $F_{i}^{+}$ and $F_{i}^{-}$ denote the positive and negative peak loads in the *i*-th cyclic loading, respectively. Based on Equations (24) and (25), Table 2 reflects the details of the health quantification of the four characteristic points of the skeleton curve in the FE simulation. Meanwhile, each working condition is established by selecting the interval process values of five safety levels corresponding to the four characteristic points during the FE simulation. This results in the stiffness-based structural health degradation curve shown in Figure 6.

### 4.3. Structural Performance Evaluation

**Figure 7:**
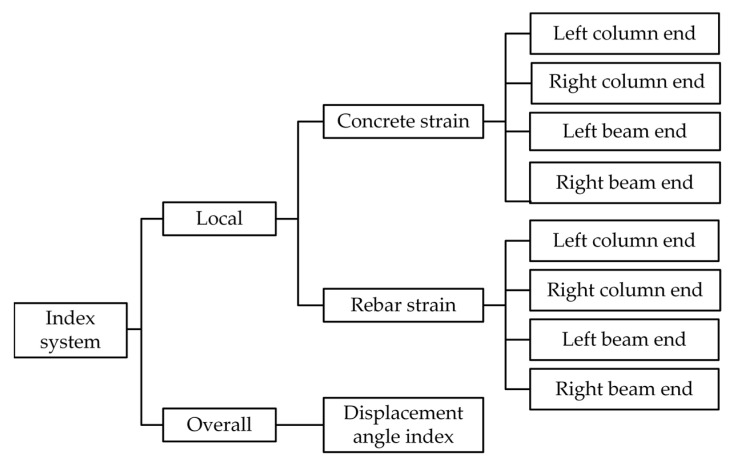
The monitoring index evaluation system of the RC frame.

In this study, both local and global performance perspectives are considered, starting from intuitive monitoring indicators. Specifically, the strains of steel rebar and concrete are selected as the local response index for components, while the inter-story drift angle at vertices is chosen as the index affecting the overall behavior of the structure. The mapping mechanism between the state index and the health degree is established using MFUCI, enabling quantification of the safety level of a particular damage state of the structure. For the local damage index, a single measurement point only reflects the localized damage degree of the structure. It is necessary to combine multi-source damage indexes and multiple monitoring measurement points to establish a comprehensive evaluation index system. The specific layout of the measurement points and the index system are shown in Figure 7.

The weight coefficients of different measurement points for the same damage index are objectively assigned by utilizing information entropy to reflect the differences in the orderliness of the information contained in each measurement point. The specific calculation is expressed as:

(26) $$R_{ij}=\frac{x_{ij}}{\sum_{i=1}^{n}x_{ij}}$$

(27) $$e_{j}=-\frac{1}{\ln(m)}\sum_{i=1}^{m}R_{ij}\ln\left(R_{ij}\ln\left(R_{ij}\right)\right)$$

(28) $$d_{j}=1-e_{j}$$

(29) $$\omega_{j}=\frac{d_{j}}{\sum_{j=1}^{m}d_{j}}$$

where *x_ij_* denotes the *i*-th sample at measurement point *j*, *R_ij_* denotes the sample ratio, *e_j_* denotes the entropy value, *d_j_* denotes the coefficient of variation, and *ω_j_* denotes the weight coefficient of the measurement point.

**Figure 8:**
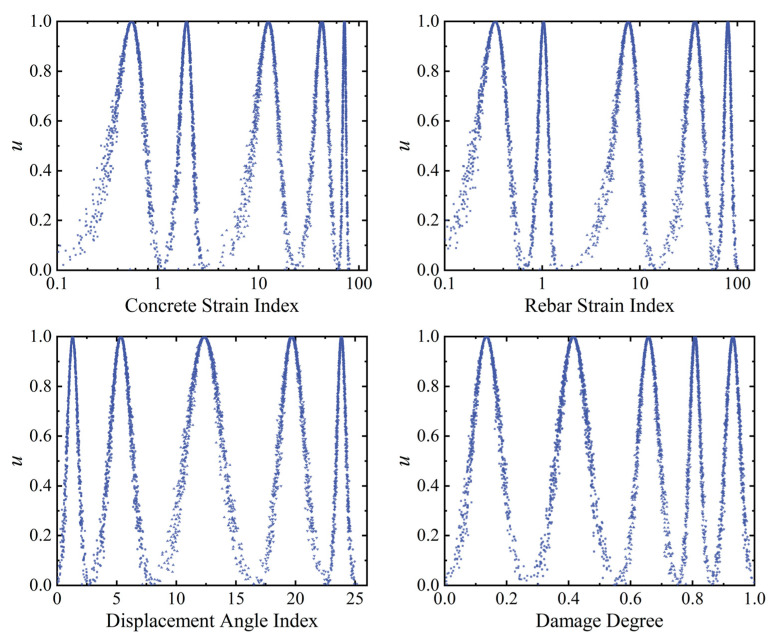
The antecedent and consequent affiliation cloud maps for the RC frame.

The state values corresponding to the damage indexes are extracted as samples in the FE. The weight coefficients for the concrete and steel rebar strains at each measurement point are solved in Equations (26)–(29), achieving the fusion of membership degrees for multiple measurement points. The response values of the damage indexes at the four characteristic states of elastic point, yield point, peak point, and ultimate point corresponding to the skeleton curve are each taken as the thresholds of the grade interval. Further, from the quantification criteria mentioned in the previous section, the *H_i_* corresponding to the four characteristic points is considered to be the grade interval limit. Based on the above, the values for damage indexes and health degree thresholds under each class interval are transformed into the respective cloud model numerical characteristics (*Ex*, *En*, *He*). Subsequently, the corresponding antecedent cloud and consequent cloud generators are constructed to establish the mapping relationship between each damage index and health degree. Table 3 presents the cloud model numerical characteristics of the damage indexes corresponding to the safety levels in the antecedent rule base, calculated using Equations (17)–(20). Similarly, Table 4 shows the cloud parameter characteristics in the consequent rule base. It is important to note that in order to comply with the computation rules of the MFUCI, the damage severity (1-*H_i_*) is used as the basis for establishing the consequent cloud, where 0 indicates that no damage has occurred in the structure. Figure 8 shows the antecedent cloud and consequent cloud generator affiliation cloud maps, individually generated for each index.

To better illustrate the performance of MFUCI, a total of five baseline models are employed, namely (a) the Bayesian neural network (BNN) with uncertainty estimation effect, (b) a long short-term memory network (LSTM) with point estimation in the time-series domain, and the three single rule inference systems, named (c) “rebar strain-based”, (d) “displacement angle-based”, and (e) “concrete strain-based”. More specifically, the Bayesian neural network consists of two Bayesian estimation layers based on variational inference to minimize the KL divergence between the variational distribution and the true posterior distribution, thereby approximating the true distribution. The LSTM minimizes the prediction error between the point estimate and the target value, and it stacks dropout layers with a 0.2 ratio to prevent overfitting. Both adopt the Adam optimizer with a 0.01 learning rate, and the epoch element is set to 100. Quantifying and comparing the structural safety evaluation capabilities of the models involves the use of accuracy, the root mean square error (RMSE), the R^2^ coefficient of determination (R^2^), and the mean absolute percentage error (MAPE). These are calculated as follows:

(30) $$RMSE=\sqrt{\frac{1}{n}\sum_{i=1}^{n}{\left({\hat{H}}_{i}-H_{i}\right)}^{2}}$$

(31) $$R^{2}=1-\frac{\sum_{i=1}^{n}{\left({\hat{H}}_{i}-H_{i}\right)}^{2}}{\sum_{i=1}^{n}{\left(H_{i}-\bar{H}\right)}^{2}}$$

(32) $$MAPE=\frac{1}{n}\sum_{i=1}^{n}\left|\frac{{\hat{H}}_{i}-H_{i}}{H_{i}}\right|$$

where ${\hat{H}}_{i}$ is the inference result, *H_i_* is the true value, and an R^2^ closer to 1 indicates a better mapping level.

**Figure 9:**
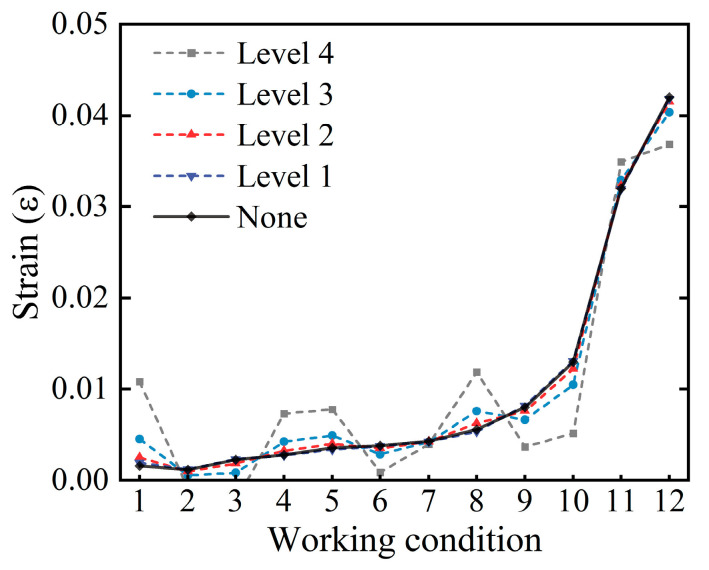
Trends in concrete strain signal curves at different noise levels.

**Figure 10:**
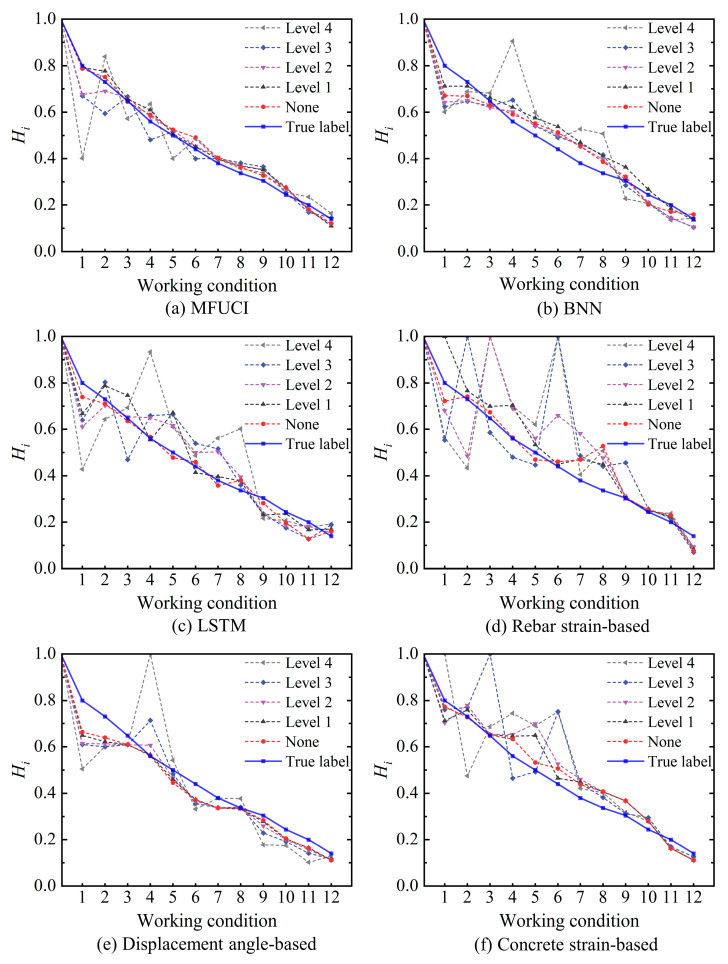
Comparison of the degradation curves of the proposed method and the baseline models in the RC frame for (**a**) MFUCI, (**b**) BNN, (**c**) LSTM, (**d**) rebar strain-based, (**e**) displacement angle-based, and (**f**) concrete strain-based.

To showcase the benefits of end-to-end modeling, this study employs sensor signals that are easy to monitor as inputs. However, the acquisition of signals is susceptible to noise interference from the routine operation and maintenance (O&M) of civil structures. This would be considered a significant source of uncertain information. Therefore, it is proposed that different levels of noise are injected into the physical model to incorporate uncertainty. It is noteworthy that in order to evaluate the noise robustness of the method, the original signals derived from the physical model are retained as the architectural set to build the corresponding cloud model architecture, while additional noise is introduced only in the inference samples. Similarly, the BNN and LSTM baseline models align with MFUCI by taking the signal as the training set and injecting only additional noise into the testing set. Different values of signal-to-noise ratio (SNR) are taken to quantify the noise level. There are four noise levels set to reveal the noise sensitivity of the model, namely, Level 1 (SNR = 40 dB), Level 2 (SNR = 30 dB), Level 3 (SNR = 20 dB), and Level 4 (SNR = 10 dB). Taking the measurement point at the right end of the concrete beam as an example, Figure 9 shows the trend of the strain signal curve at different noise levels. As the noise intensifies, there is a degree of signal drift and turbulence which reflects the uncertainty. Based on the multi-source fusion generator built from the original signals derived from the physical model, samples of the noise-added signals are fed into the MFUCI, demonstrating the RMSE, R^2^ scores and MAPE comparisons of the proposed method and baseline models, as shown in Table 5. Compared to the baseline models, the proposed MFUCI exhibits superior performance with lower RMSE and MAPE and better R^2^ scores for different noise levels. In detail, the sampled health degradation curves for the MFUCI and baseline models at each noise level are plotted in Figure 10. In the baseline models, LSTM demonstrates quite competitive results with MFUCI at the noiseless level, benefiting from the powerful point-estimation backpropagation algorithm. However, with the injection of uncertainty, BNN overtakes LSTM in terms of uncertainty estimation effects. This occurs because BNN approximates the posterior distribution rather than a specific target value. In parallel, the rebar strain-based and concrete strain-based inference results in the noiseless background being more accurately determined in the early stage of damage, while the inference fluctuates more as the nonlinear damage of the structure increases. With the injection of noise, both have limited uncertainty estimation capabilities. The inference is more stable when based on the displacement angle alone rather than on both types of strain, but the error is larger when the structure is intact. Benefiting from multi-source data fusion, the proposed MFUCI outperforms the baseline models with respect to stability and robustness against uncertain data under different noise levels, with a slight decrease with higher noise levels. Based on the above, the proposed method applied to a safety evaluation at the structural level demonstrates competitive structural safety quantification capabilities.

## 5. RC T-Beam Damage Experiment

This section focuses on two RC T-beam experiments in which the relationship between the condition indexes and the stiffness level of the components was analyzed to validate the effectiveness of MFUCI for safety evaluations at the component level.

### 5.1. Experiment Description

**Figure 11:**
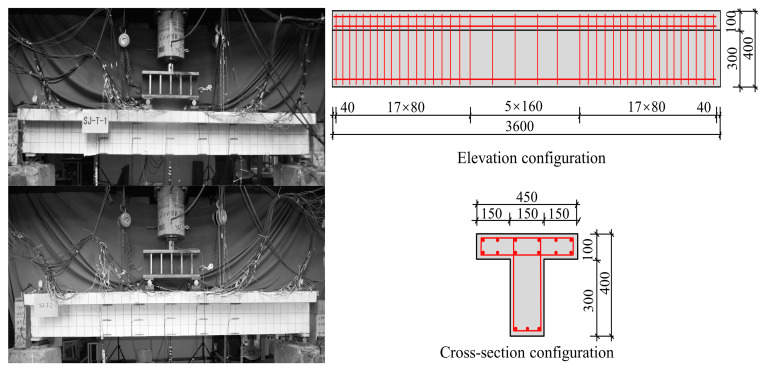
The RC T-beam components and the reinforcement diagrams.

As shown in Figure 11, the two reinforced RC T-beams (SJ-T-1 and SJ-T-2) have a length of 3600 mm and a section height of 400 mm, where the flange thickness is 100 mm with a width of 450 mm, and the web height is 300 mm with a width of 150 mm. Two layers of rebar mesh with 12 longitudinal bars of 8 mm in diameter are set in the flange. The bottom of the web has three longitudinal bars of 14 mm in diameter. The deflection measurement points were arranged at the quartile (W-1 and W-3), mid-span (W-2), and supports (Z1 and Z2) and measured using LVDT displacement meters. The strain gauges in the concrete were arranged at the top, web, and bottom of the T-beams. The rebar strain gauges were arranged at the flange plate of the T-beams and the bottom rebars of the web.

**Figure 12:**
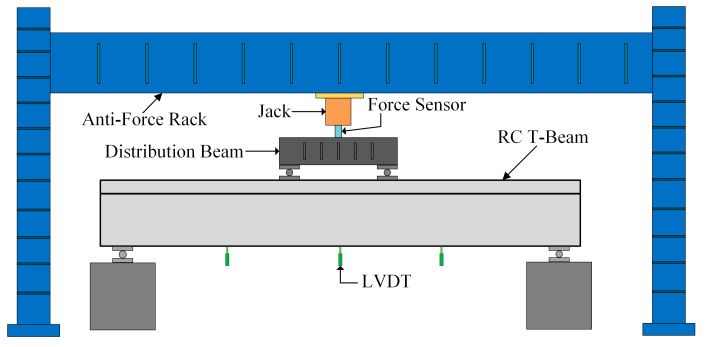
The RC T-beam configuration details.

A schematic diagram of the experimental loading is shown in Figure 12. Top-down loading via a jack was used to realize the loading and unloading process by applying the load to the distribution beam and then transferring from the distribution beam to the T-beams. Further, the pressure sensor was set between the jack and the distribution beam to measure the magnitude of the loading value, which was automatically collected by the computer through the acquisition system.

The experimental loading process is divided into pre-loading and four formal loadings. The specific loading process is as follows:

1. Pre-loading: Load to 15 kN in 5 kN increments and unload to 0 kN. Observe specimen, device, and instrumentation to ensure proper operation and timely troubleshooting;
2. First loading: Load in 5 kN increments until the specimen reaches crack loading and then unload to 0 kN;
3. Second loading: Load to crack loading in 5 kN increments. Subsequently, load to 50 kN in 10 kN increments and then unload to 0 kN;
4. Third loading: Load to 80 kN in 10 kN increments and then load to 0 kN;
5. Fourth loading: Force-controlled loading is initially applied in 10 kN increments. When the slope of the load–displacement curve at the mid-span appears to decrease, there is a change to mid-span displacement-controlled loading with 2 mm per level until the specimen is damaged.

### 5.2. Experimental Results and Damage Analysis

**Figure 13:**
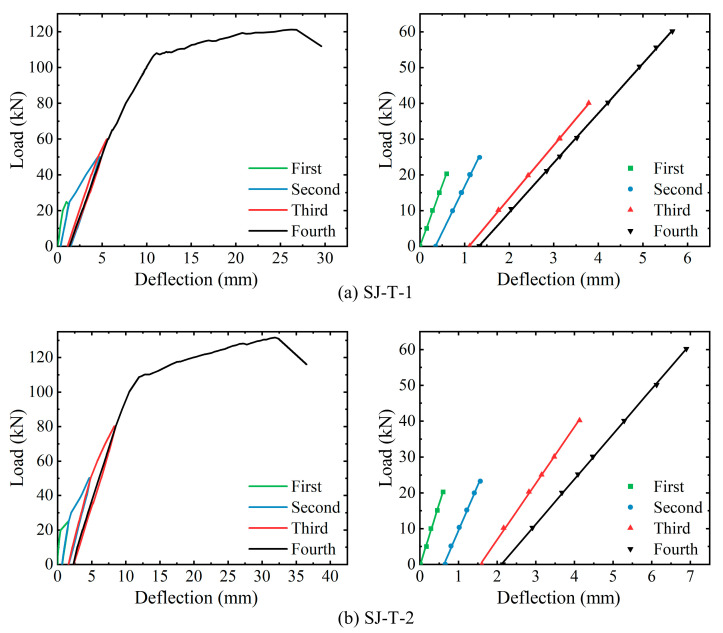
Analysis of load–displacement curves for SJ-T-1 and SJ-T-2.

Both the SJ-T-1 and SJ-T-2 loading experiments were conducted with four formal loads, and each load was reduced to 0 kN after the further development of damage. The load–displacement curves at the mid-span sections of the two reinforced RC T-beams and the linear fitting curves in the elastic phase are shown in Figure 13. Components SJ-T-1 and SJ-T-2 in the load–displacement curves exhibit a precise change trend, and all four loadings show linear growth at the beginning. As the cracks continue to develop during each loading, the slope of the linear phase gradually decreases, and the stiffness continuously degrades.

**Figure 14:**
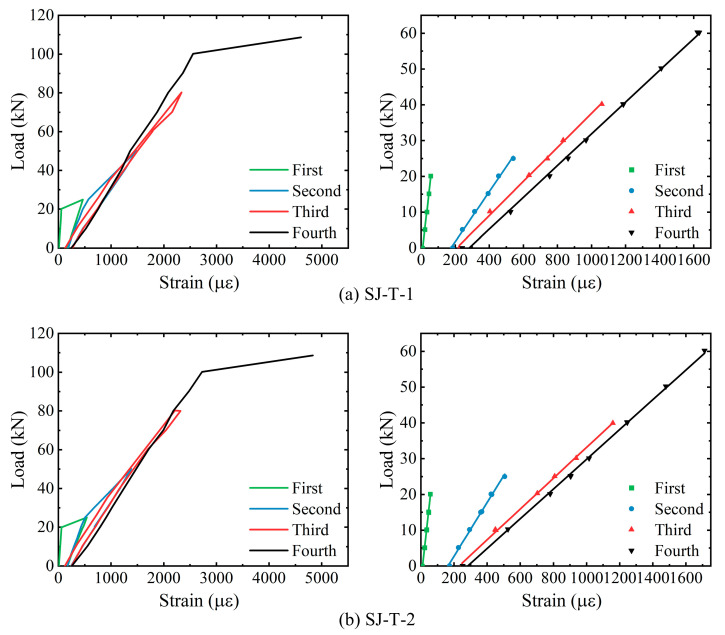
Analysis of load–strain curves for rebars at bottom of SJ-T-1 and SJ-T-2.

**Figure 15:**
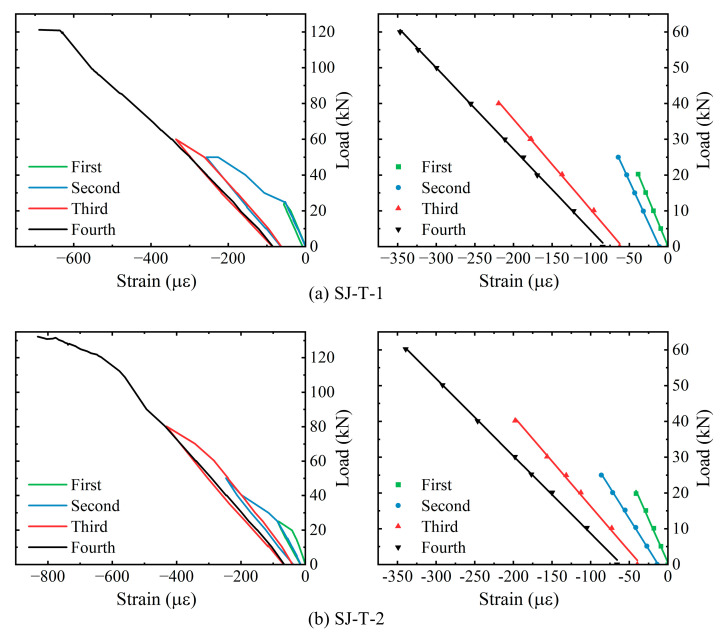
Analysis of load–strain curves for concrete in the pure bending section of SJ-T-1 and SJ-T-2.

Table 6 shows the cracking loads, ultimate loads, and the corresponding mid-span vertical displacements for each of the components, i.e., SJ-T-1 and SJ-T-2. The corresponding load–strain curves, as well as the linear fitting curves of the elastic phase, are derived for strain measurement points N4-8 of the spanwise section within the purely bending section of rebar N4 at the bottom of components SJ-T-1 and SJ-T-2, respectively, as shown in Figure 14. The yielding of the tensile rebars at the mid-span section of both components occurs before the fourth loading. The overall trend of strain variation with load at the purely bending sections of components SJ-T-1 and SJ-T-2 remains consistent, while the slope of the load–strain curve continuously decreases during the four loading sessions. The corresponding load–strain curves of concrete strain measurement point in the span of the top slab within the purely curved sections of components SJ-T-1 and SJ-T-2, and the linear fitting curves of their respective elastic phases, are shown in Figure 15. According to the curves, the trend of concrete compressive strain with load at the measurement points remains consistent.

According to the above analysis results, the trends of vertical displacement in the span, compressive concrete strain at the span cross-section, and tensile steel strain at the bottom span cross-section are similar for components SJ-T-1 and SJ-T-2 during loading and unloading. At the beginning of each loading, the mid-span displacement, tensile rebars, and compressive concrete strain increase linearly with increasing load. Concurrently, due to the continuous development of component damage, there is a decreasing trend for the linear phase of all three monitoring indexes compared to the previous loading. Therefore, the proposed study focuses on the relationship between each damage condition index and the performance level under four linear loading stages.

### 5.3. Construction of MFUCI

By comparing the experimental results of SJ-T-1 and SJ-T-2, it can be observed that certain deviations exist but with the same trend between the outcomes of the two specimens due to uncertainties such as the non-uniformity of the material, monitoring errors, and initial defects in the model. These uncertainties manifest as fuzziness and uncertainty in the condition indexes and damage extent. In this regard, the advantages of the robustness of the proposed method in the face of uncertain data are demonstrated using a form of cross-domain inference. Specifically, the samples of component SJ-T-2 are considered architecture sets to build a cloud inference qualitative rule base, while the samples of component SJ-T-1 are considered inference sets for health degree mapping inference. According to the force characteristics of reinforced concrete, the mid-span deflection, the strain of rebars in the tensile zone at the bottom purely bending section of the beam, and the strain of concrete in the compressive zone at the top are selected as the three condition input parameters for MFUCI. According to our analysis in the previous section, spanwise deflection, tensile rebar strain, and compressive concrete strain within the purely bending section of the component are found to exhibit different rates of linear growth under different loading stages. Consequently, the magnitude of the rate of change of each damage index is used as an input parameter for MFUCI in this paper. To eliminate the effect of static load, the rate of change of each index under load is defined to achieve MFUCI universality:

(33) $$\left\{\begin{array}{c} \Delta_{i}^{1}=\frac{w_{i}-w_{0}}{F_{i}}\\ \Delta_{i}^{2}=\frac{\varepsilon_{i}^{t}-\varepsilon_{0}^{t}}{F_{i}}\\ \Delta_{i}^{3}=\frac{\varepsilon_{i}^{c}-\varepsilon_{0}^{c}}{F_{i}} \end{array}\right.$$

where parameters $w_{i}$, $\varepsilon_{i}^{t}$, and $\varepsilon_{i}^{c}$ denote the deflection, tensile rebar strain, and compressive concrete strain values, respectively, when a static load is applied to the component, $w_{0}$, $\varepsilon_{0}^{t}$, and $\varepsilon_{0}^{c}$ denote the initial values, and $F_{i}$ denotes the magnitude of the applied load.

Typically, the component suffers damage that manifests as a stiffness degradation phenomenon. In practice, the damage index of a component is defined as the relative reduction in stiffness of the specimen measured in a mechanical test [50,51,52]. Combined with the definition of the health degree given in Equation (22), it is specified as:

(34) $$H_{i}=\frac{K_{e,i}}{K_{e,0}}$$

where *K*_e,0_ is the initial stiffness of the component and *K*_e,*i*_ is the elastic stiffness of the specimen in the reloading stage. The health results corresponding to the loading stages of components SJ-T-1 and SJ-T-2 are shown in Table 7.

**Figure 16:**
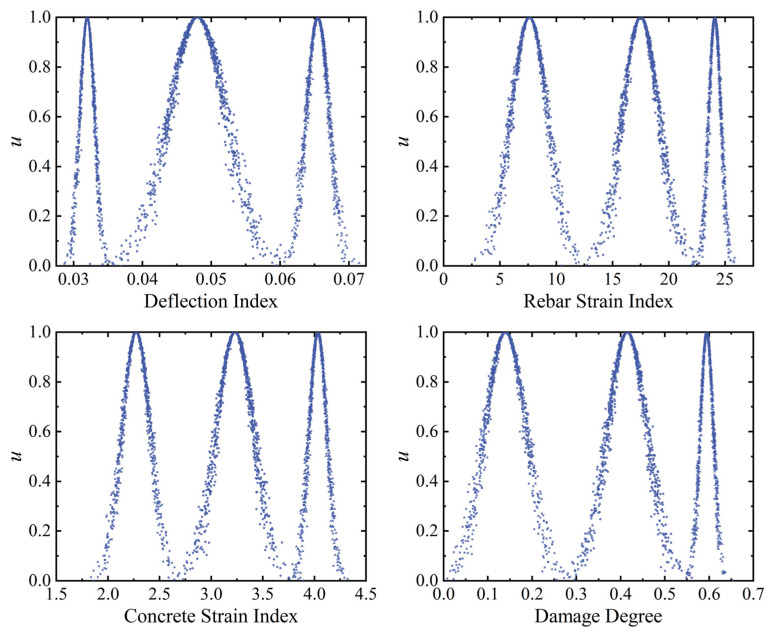
The antecedent and consequent affiliation cloud maps for the RC T-beams.

Above, the indexes and health degrees under all loading levels for the loading stages of component SJ-T-2 are used as architecture sets to construct specific qualitative rules using the multi-source fusion generator. Specifically, the mid-span deflection, rebar strain, and concrete strain monitoring values corresponding to all loading levels under the damage states of component SJ-T-2 are selected as the basis for establishing the antecedent rule base. According to Equation (33), the damage index values corresponding to the monitoring data under each working condition of component SJ-T-2 are calculated. Further, each damage index under the four working conditions is graded as a threshold value for each of the three grade intervals. As shown in Table 8, the cloud model parameters (*Ex*, *En*, *He*) are calculated for each damage modality indicator, corresponding to each safety level according to Equations (17)–(20). To comply with the rules for calculating the consequent cloud parameters, the damage degree corresponding to each working condition is used as the basis for the consequent cloud. Specifically, 1-*H_i_* is the consequent cloud rule, where *H_i_* is the health value corresponding to component SJ-T-2 in each damage state; the larger the value of 1-*H_i_*, the more severe the damage to the component. The rules for the characterization of the consequent cloud and the parameters of the cloud model are shown in Table 9. Figure 16 shows the generation of each index by the antecedent cloud generator and the consequent cloud generator affiliation cloud maps.

### 5.4. Inference Results

**Figure 17:**
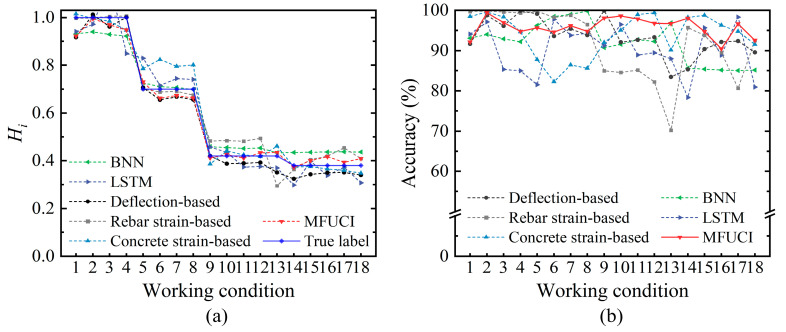
Comparison of the (**a**) degradation curves and (**b**) accuracy between the proposed method and the baseline models with samples of SJ-T-1 as the inference sets.

In the previous section, the experimental data for component SJ-T-2 are considered as the architecture sets to build an MFUCI-based qualitative rule base. Considering the effects of monitoring noise as well as the non-uniformity of concrete materials, the experimental data of SJ-T-1 are fed into MFUCI as cross-domain inference samples to demonstrate the robustness and accuracy of the proposed method. Table 10 shows the performance metrics of the proposed study in comparison with baseline models. It is observed that the proposed approach demonstrates superior performance in component health degree mapping compared to the baseline models, with lower RMSE, MAPE, and improved R^2^ score. Concretely, Figure 17 is a comparative illustration of the sampled health degradation curves and mapping accuracy for the MFUCI and baseline models. It is observed that the maximum error of health prediction obtained when considering the deflection index as an input parameter is 16.57%. The deflection index of component SJ-T-1 in the intact and early stages of damage can better reflect the health level. With increasing damage and late measurement errors, the health prediction is significantly biased. The health predictions obtained with the tensile rebar strain index as an input parameter have greater fluctuation, with a maximum error of 29.79%. However, the inference accuracy exceeds 99% in the uncracked and early damage development of the components. Compared to the deflection and rebar strain indexes, the maximum error in the prediction of health based on the concrete strain damage index is 17.71%, but there is still instability due to errors in a single index. In contrast, MFUCI, which considers the fusion of multiple sources of damage metrics, has a high mapping capability for the inference samples of component SJ-T-1, with an accuracy consistently above 90%. On the other hand, the BNN model exhibits a prediction accuracy second only to the proposed method facing cross-domain inference forms, due to its uncertainty estimation capability. However, the LSTM only performs deterministic point estimation on the training data and shows a lack of ability to generalize cross-domain data with uncertainty. Moreover, the proposed method does not involve a gradient computation and back propagation process, as in BNN and LSTM, and only utilizes the architectural sets to build the architecture of the model, thus demonstrating a better safety evaluation capability in the context of scarce and limited damaged samples. Synthetically, MFUCI provides a better reflection of the current health status at the component level.

## 6. Conclusions

This paper presents a novel structural safety evaluation method, named multi-source fusion uncertainty cloud inference, to address the uncertainties arising from initial defects and monitoring errors in practical engineering structures. Leveraging the advantages of cloud modeling theory in handling uncertainty in knowledge, this method provides a comprehensive approach to assessing structural safety. Focusing on RC structures, this study investigates methods of safety evaluation from the component level to the structural level. The evaluation is conducted through experimental damage tests on RC components and FE simulations of a single-story RC frame structure. By considering both the individual components and the entire structure, a comprehensive evaluation of safety is achieved. Based on the above work, the conclusions of this paper are as follows:

1. This study is focused on investigating the relationship between characterizing condition indexes and structural performance to quantitatively evaluate the structural health status. Considering the influence of multi-source data in structural safety evaluations, a multi-source fusion uncertainty cloud inference architecture is proposed as a theoretical basis for quantifying the structural safety degree;
2. A single-story RC structure was investigated for damage under low-cycle reciprocating loads. The safety level was quantified by extracting characteristic points from the skeleton curve, and the effectiveness of the proposed approach in conducting safety evaluations at the structural level was validated;
3. Damage experiments on two RC T-beams were conducted to analyze the failure process in terms of the specimen condition index and specimen stiffness degradation. A safety evaluation system for reinforcement strain, concrete strain, and deflection was developed based on the proposed MFUCI, demonstrating that it is suitable for safety evaluations at the component level;
4. Considering the variations of civil building O&M environments, the model was tested for different levels of immunity by injecting uncertainty information, i.e., adding noise only to the inference samples. The results show that the proposed model has excellent noise immunity under different noise levels;
5. Considering the non-uniformity of the material and the influence of the fabrication process of the components, the excellent generalizability and robustness of the proposed study are demonstrated by using SJ-T-2 as the basis of the architecture and SJ-T-1 as the inference samples in RC T-beams.

When dealing with a complicated structural system using the proposed method, it is suggested that the system be divided into substructural systems. On the one hand, the proposed method permits the direct evaluation of critical substructure systems, thus facilitating evaluations of critical components. On the other hand, each substructure is considered an information source when employing multi-source fusion inference to evaluate the whole structure. The critical point is to apply representational signals that can be easily and directly monitored by the in-service structure as local condition indexes. In future work, we will focus on developing a digital twin framework for finite element synergy, where a physically synergistic finite element model is taken as the architectural set of the proposed method, while the response signals of the real structure are taken as the inference set to accurately evaluate the in-service structure.

## Figures and Tables

**Table 1:** Cloud drops: contribution.

Cloud Drop Group	Universe Interval	Contribution
Backbone element	[Ex−0.67En,Ex+0.67En]	50.00%
Basic element	[Ex−En,Ex+En]	68.26%
Peripheral element	[Ex−2En,Ex−En]∪[Ex+En,Ex+2En]	27.18%
Weak peripheral element	[Ex−3En,Ex−2En]∪[Ex+2En,Ex+3En]	4.30%

**Table 2:** Details of the health quantification of the four characteristic points of the skeleton curve in the FE simulation.

Characteristic	$F_{i}^{+}(\mathrm{kN})$	$F_{i}^{-}(\mathrm{kN})$	$\Delta_{i}^{+}/\Delta_{i}^{-}(\mathrm{mm})$	$H_{i}$
Elastic point	16.145	−15.579	±4	0.734
Yield point	29.658	−27.736	±12	0.443
Peak point	33.710	−32.392	±25	0.245
Ultimate point	28.832	−24.097	±34	0.144

**Table 3:** The antecedent rule base cloud parameters for the RC frame.

Grade	Parameter	Concrete Strain Index	Rebar Strain Index	Displacement Angle Index
Ⅰ	*Ex*	0.550	0.330	1.335
	*En*	0.183	0.110	0.450
	*He*	0.018	0.011	0.045
Ⅱ	*Ex*	1.930	1.030	5.335
	*En*	0.277	0.123	0.888
	*He*	0.028	0.012	0.089
Ⅲ	*Ex*	12.58	7.650	12.35
	*En*	3.273	2.083	1.450
	*He*	0.327	0.208	0.145
Ⅳ	*Ex*	42.70	36.95	19.70
	*En*	6.767	7.683	1.000
	*He*	0.677	0.768	0.100
Ⅴ	*Ex*	71.50	80.00	23.85
	*En*	2.833	6.667	0.383
	*He*	0.283	0.667	0.038

**Table 4:** The consequent rule base cloud parameters for the RC frame.

Grade	1-*H_i_*	*Ex*	*En*	*He*
Ⅰ	(0.00, 0.27)	0.1350	0.0450	0.0045
Ⅱ	(0.27, 0.56)	0.4150	0.0480	0.0048
Ⅲ	(0.56, 0.76)	0.6580	0.0327	0.0033
Ⅳ	(0.76, 0.86)	0.8080	0.0173	0.0017
Ⅴ	>0.86	0.9300	0.0233	0.0023

**Table 5:** Comparison of performance metrics in the RC frame at four noise levels using the proposed method and baseline models.

Model	Noise Level	RMSE	R^2^	MAPE
Rebar strain-based	None	0.0693	0.8841	0.1431
	Level 1	0.0868	0.8290	0.1549
	Level 2	0.1660	0.3856	0.2773
	Level 3	0.2063	0.0459	0.3337
	Level 4	0.2324	−0.1748	0.3439
Displacement angle-based	None	0.0592	0.9191	0.1137
	Level 1	0.0635	0.9072	0.1168
	Level 2	0.0746	0.8726	0.1348
	Level 3	0.0911	0.8082	0.1682
	Level 4	0.1694	0.3167	0.2591
Concrete strain-based	None	0.0478	0.9452	0.1245
	Level 1	0.0681	0.8899	0.1497
	Level 2	0.0851	0.8318	0.1779
	Level 3	0.1422	0.5443	0.1938
	Level 4	0.1537	0.4774	0.2464
BNN	None	0.0581	0.9180	0.1205
	Level 1	0.0613	0.9087	0.1243
	Level 2	0.0666	0.8922	0.1401
	Level 3	0.0765	0.8577	0.1601
	Level 4	0.1402	0.5225	0.2436
LSTM	None	0.0361	0.9683	0.0986
	Level 1	0.0764	0.8583	0.1353
	Level 2	0.0861	0.8199	0.1637
	Level 3	0.1101	0.7052	0.2454
	Level 4	0.1862	0.1572	0.3182
MFUCI	None	0.0257	0.9839	0.0688
	Level 1	0.0299	0.9784	0.0789
	Level 2	0.0449	0.9511	0.0881
	Level 3	0.0651	0.8979	0.1049
	Level 4	0.1286	0.6009	0.1505

**Table 6:** Cracking and ultimate load details of SJ-T-1 and SJ-T-2.

Component	Stage	Load (kN)	Mid-Span Deflection (mm)
SJ-T-1	Concrete cracking	25	1.21
SJ-T-1	Ultimate state	121	26.81
SJ-T-2	Concrete cracking	25	1.61
SJ-T-2	Ultimate state	132	32.29

**Table 7:** The health degrees corresponding to each loading phase of SJ-T-1 and SJ-T-2.

Component	Configuration	State 1	State 2	State 3	State 4
SJ-T-1	Inference sets	1.00	0.70	0.42	0.38
SJ-T-2	Architecture sets	1.00	0.72	0.45	0.36

**Table 8:** The antecedent rule base cloud parameters for the architecture sets of SJ-T-2.

Grade	Parameter	Deflection Index	Rebar Strain Index	Concrete Strain Index
Ⅰ	*Ex*	0.0320	7.6200	2.2740
	*En*	0.0011	1.6410	0.1380
	*He*	0.0001	0.1641	0.0138
Ⅱ	*Ex*	0.0480	17.492	3.2310
	*En*	0.0042	1.6493	0.1810
	*He*	0.0004	0.1649	0.0181
Ⅲ	*Ex*	0.0655	24.086	4.0350
	*En*	0.0017	0.5490	0.0870
	*He*	0.0002	0.0549	0.0087

**Table 9:** The consequent rule base cloud parameters for the architecture sets of SJ-T-2.

Grade	1-*H_i_*	*Ex*	*En*	*He*
Ⅰ	(0.00, 0.28)	0.1400	0.0467	0.0047
Ⅱ	(0.28, 0.55)	0.4150	0.0450	0.0045
Ⅲ	>0.55	0.5950	0.0150	0.0015

**Table 10:** Performance comparison of the proposed method and baseline models inference results with samples of SJ-T-1 as the inference sets.

Model	RMSE	R^2^	MAPE
Rebar strain-based	0.0488	0.9606	0.0828
Deflection-based	0.0401	0.9737	0.0669
Concrete strain-based	0.0529	0.9538	0.0620
BNN	0.0477	0.9622	0.0799
LSTM	0.0713	0.9156	0.0973
MFUCI	0.0313	0.9837	0.0419

## Data Availability

The data analyzed during this study are available from the corresponding author upon reasonable request with respect to ethical and privacy restrictions.
